# Statistics in Reference to the Frequency of Tuberculosis in Animals Slaughtered at Augsburg in 1883*Adam’s Wochenschrift für Thierheilkunde, No. 15, 1884.

**Published:** 1884-07

**Authors:** 


### STATISTICS WITH REFERENCE TO THE FREQUENCY OF TUBERCULOSIS IN ANIMALS SLAUGHTERED AT AUGSBURG IN 1883.*

The following remarks have not only their value as showing the frequency of tuberculosis among cattle and other animals slaughtered at this place, but they may be also instructive to us, showing how inspection should be done in this country. Here there are no regular veterinary inspectors at our slaughtering houses. A butcher answers the purpose. All system is wanting. All we are informed is the number of carcasses condemned, but what diseases prevail is a matter not considered, nor are any statistics kept as to their frequency. Thus much valuable information is lost to the public.

Among 30,922 swine slaughtered at Augsburg, only three cases of tuberculosis were discovered.

In 1883, 11,829 head of cattle were killed, 7,510 being males, 4,336 castrated oxen, 2,890 non-castrated steers, 284 young steers

* Adam's Wochenschrift für Thierheilkunde, No. 15, 1884.

(castrated), 4319 females, of which 3,868 were cows and 451 heifers. Of these, 371 were found tuberculous, 3.12 per cent.; 83 being males (1.10 per cent.) and 288 (6.66 per cent.) females, 21,994 calves were killed, tuberculosis being found in but one, a male, 3 months old, of the mountain breed.

Then follows the table of the sex and age of each infected animal. The tuberculous processes were found in the lungs and the serosæ of one or both the visceral cavities in 126 cases. In 188 cases the lungs only were infected, the serosæ being free. In 53 cases the serosæ alone were infected ; in 4 the liver was the seat of the disease.

Then comes a table giving breed, cross, place of raising, etc., of the diseased animals, with remarks about the general conditions of their bodies.

